# Changes in milk composition of raw, frozen, pasteurized, and freeze-dried jenny milk from a farm-scale dairy

**DOI:** 10.1016/j.fochx.2026.104240

**Published:** 2026-07-22

**Authors:** Le-tian Zhang, Ke Zhang, Allan Degen, Qian Xu, Jiang-lin Xiong, Xiao-ling Zhou

**Affiliations:** a College of Animal Science and Technology, Tarim University, Key Laboratory of Livestock and Grass Resources Utilization around Tarim, Ministry of Agriculture and Rural Areas, Key Laboratory of Tarim Animal Husbandry Science and Technology, Xinjiang Production & Construction Corps, Alar, Xinjiang 843300, China; b Desert Animal Adaptations and Husbandry, Wyler Department of Dryland Agriculture, Blaustein Institutes for Desert Research, Ben-Gurion University of the Negev, Beer Sheva 8410500, Israel; c College of Food Science and Technology, Tarim University, Alar, Xinjiang 843300, China; d Hubei Key Laboratory of Animal Nutrition and Feed Science, School of Animal Science and Nutrition Engineering, Wuhan Polytechnic University, Wuhan 430023, China

**Keywords:** Jenny milk, Metabolome, Trace element, Fatty acid, Pasteurized milk, Lyophilized milk

## Abstract

Jenny milk is prone to deteriorate and is intolerant of freezing and heating. This study compared proximate components, metabolomes, trace elements, and fatty acids (FAs) in raw (RM), frozen (DM), pasteurized (PM), and freeze-dried (FM) jenny milk from milking to final products. Proximate components in RM were lower than the others. Compared with RM, differential metabolites in DM, PM, and FM were associated with protein, amino acid, nucleotide, and carbon metabolism, whereas differences among DM, PM, and FM were related to unsaturated lipid metabolism. Trace element concentrations varied during processing, but toxic elements remained within safety limits in all samples. DM had lower levels of MUFA, n-6 PUFA, and n-9 UFA than FM and PM, while total FA content did not differ among milk types. Therefore, the time between milking and freezing should be shortened and processing technology should be modified to improve the fidelity of jenny milk.

## Highlights

• Lipid metabolites in pasteurized and lyophilized jenny milk were mainly affected.
• Lyophilized jenny milk has higher concentrations of Co, Mn, Zn, Ni, Cr and As.
• Pasteurized and lyophilized jenny milk had lower percentage of MCFA and higher percentage of LCFA.
• Lyophilized jenny milk has higher percentage of MUFA, n-6 PUFA, and n-9 UFA.

## 1 Introduction

Jenny milk is low in fat content but rich in whey protein, lactose, unsaturated fatty acids (UFA), essential minerals and vitamins (Wei et al., 2025; Živkov Baloš et al., 2023). The milk is characterized by low allergenicity and possesses anti-inflammatory (Cai et al., 2025; Huimin et al., 2025), antioxidant (Trinchese et al., 2021), and anti-type II diabetes (Li et al., 2020) properties. Nutritionally, it is considered a healthy food, and is prescribed widely for children with compromised immunity, the elderly, and people with health problems (Xu et al., 2025). The global production of jenny milk has been increasing recently (Meena et al., 2024), but the quality and safety of the milk products needs more attention, which could be a key factor in the sustainable development of the jenny milk industry.

Milk is a viscous colloidal fluid, and its components are influenced by such factors as containers, transportation, storage conditions, circulation pipelines, processing equipment and packaging materials (Gao et al., 2019; Rabbani et al., 2025; Walczak-Skierska et al., 2024). According to our observations, dairy donkey farms in China predominantly house more than 200 heads and most employ the trolley-type or fishbone-style machine for milking. Jenny milk products include mainly pasteurized liquid milk and lyophilized milk powder (Seyiti & Kelimu, 2021). It was reported that proximate components, fatty acids (FA), and mineral elements in jenny milk were not affected by lyophilization (Polidori et al., 2019) or pasteurization (Martini et al., 2018), but there was a decrease in lactose percentage and variations in some FA at 90 d of freezing (Martini et al., 2018). Improper processing temperatures can cause the denaturation of whey protein in jenny milk and alter the colloidal properties (Luo et al., 2019), thus affecting biological activities (Cai et al., 2025; Tedeschi et al., 2023). Heat can affect the contents of total protein, casein, whey protein, and lysozyme in jenny milk (Polidori & Vincenzetti, 2013). However, little is known about the effect of milking, storage and processing on milk components and metabolome of jenny milk.

FA in milk exist either in a free state (free FA, FFA) or are esterified into triglycerides (TG) (Wei et al., 2025), which divide mainly into short-chain FA (SCFA) with 1 to 6 carbon atoms, medium-chain FA (MCFA) with 8 to12 carbon atoms, and long-chain FA (LCFA) with 14 to 24 carbon atoms. Changes in FA are important indicators for monitoring and evaluating the hygiene of farm and milking equipment, and the freshness and quality of milk (González-Córdova & Vallejo-Cordoba, 2003; Woodhouse et al., 2025), as they can affect the oxidative stability and shelf life of dairy products (Antonelli et al., 2002; Talbot, 2016). Some FFA are inherently present in milk, and some are formed after the degradation of milk fat (Deeth, 2020). Milk fat degradation is divided into spontaneous, bacterial and induced lipolysis. Spontaneous lipolysis is catalyzed by lipoprotein lipase and, therefore, is affected by the physiological state of the dairy animal and the temperature of the raw milk (Rabbani et al., 2025). Bacterial lipolysis is catalyzed by bacterial lipases and is affected by hygiene conditions during milking and cooling, while induced lipolysis results from the mechanical action on the milk fat globule membrane during transportation and processing (Woodhouse et al., 2025). The FFA and the secondary metabolites such as ketones, aldehydes, and esters influence the flavor of dairy products (Yan et al., 2024). Factors influencing the FA composition in jenny milk include animal factors (breed, genetics, parity, lactation stage) (Martemucci & D'Alessandro, 2012; Wu et al., 2025) and feeding management (Valle et al., 2018). Studies on changes in FFA and FA of jenny milk during processing are lacking.

Trace elements such as iron (Fe), zinc (Zn), iodine (I), selenium (Se), copper (Cu), cobalt (Co), manganese (Mn), and molybdenum (Mo) are widely involved in body metabolism, immunity and neuromodulation, and are indispensable for human health (Mehri, 2020; Rolić et al., 2025). The concentrations of the trace elements Fe, Zn, and Cu in raw jenny milk were reported (Ljubojević Pelić et al., 2025; Malacarne et al., 2019), however, the impact of storage and processing on essential trace elements in jenny milk remains unknown. Elements of lead (Pb), cadmium (Cd), mercury (Hg), and arsenic (As) are potentially toxic as they can damage the nervous system, skeleton, digestive tract, kidneys, circulation, and immunity functions (Alinezhad et al., 2024; Mehri, 2020). Some countries and regions, such as the European Union (Directorate-General for Health and Food Safety of European Commission, 2023), the United States (National Conference on Interstate Milk Shipments, 2023), and China (Ministry of Health of the People's Republic of China, 2025), have set upper safe limits for the concentrations of Pb, Hg, As, and chromium (Cr) in dairy products. The concentrations of As, Pb, Hg and Cd in raw jenny milk are generally within the safe range (Ljubojević Pelić et al., 2025), however, the effect of storage, transportation, and processing on toxic elements of jenny milk remains uncertain.

Previous studies examined the effects of heat or cold storage on the proximate composition, protein fractions, FA profiles, and mineral elements of jenny milk under laboratory conditions (Luo et al., 2019; Martini et al., 2018; Polidori et al., 2019), and focused on an individual processing step or on specific components. The sequential changes in jenny milk composition during industrial production, that is, from milking to storage to processing are critical and of interest to both consumers and producers. In the present study, composition, essential and toxic trace elements, untargeted metabolomics, and FA profiling were determined in raw, frozen, pasteurized, and freeze-dried jenny milk. This study aimed to clarify the dynamic alterations in composition from milking to storage to processing jenny milk, which could provide a basis for quality control and safety of jenny milk.

## 2 Materials and methods

All procedures on the jennies were approved by the Animal Ethics Committee of the University of Tarim (2022003), China. Mandatory laboratory health and safety procedures were followed as outlined in the operation guide manual of experimental work.

### 2.1 Site, jennies and experimental design

The jennies were maintained and milked, and the milk was processed in a commercial farm-scale dairy in Kuqa, Xinjiang, China using. The farm raises over 1000 jennies, including approximately 200 lactating jennies year-round. Lactating jennies are hand-milked twice daily at 11:00 and 19:00 in a fishbone-style milking parlor at 20 °C to 25 °C. The schedule of milking and milk processing was as follows: following milking, the raw milk was frozen and was maintained for a short-term in a depository. Once enough milk was accumulated for batch processing, the milk was pasteurized to produce milk and milk products and, subsequently, freeze-dried under vacuum to obtain milk powder. Thus, fresh raw milk from individual jennies was pooled and tracked from the same production day, and this pooled milk was stored frozen in a depository, pasteurized and freeze-dried, which represented the key quality control points during processing. In this study, we used a longitudinal processing-chain batch-tracking design so as not to interfere with the normal milking and milk processing of the dairy.

### 2.2 Sample collection

On May 2nd, 2024, seven jennies at the mid-lactation stage (90 +/− 10 days) were selected randomly and 100 mL of raw milk (**RM**) were collected after discarding the initial 20 mL of milk. Each sample from the morning and evening milkings was mixed well and divided into five aliquots: one for the determination of proximate components, and the others were stored at −20 °C for further analyses.

Within 1 to 2 h after milking, pooled raw milk was transferred into clean pre-cooled SUS304 stainless steel tanks [50 L capacity; the tank components consisted of iron (≥ 65%), carbon (≤ 0.08%), chromium (18% to 20%), nickel (8.0% to 10.5%), silicon (Si) (≤ 0.75%), and manganese (Mn) (≤ 2.00%)]. The milk was pre-cooled at −20 °C for 4 to 8 h, then stored in a cold depository (−15 ± 3 °C) for 3 to 4 days until pasteurization. After this depository period, eight tanks containing milk from the tracked pooled milk batch were selected randomly, and 200 mL of depository raw milk (**DM)** were collected from each tank. Each DM sample was divided into five aliquots for analysis.

Six glass bottles (soda-lime-silica glass containing 70 to 75% SiO₂, 13 to 15% Na₂O, 5 to 10% CaO, and small amounts of MgO and Al₂O₃) of pasteurized milk (**PM**), produced from the same tracked pooled milk batch, were selected randomly from the milk processing section. A 200 mL milk sample was taken from each bottle and divided into 5 aliquots for analysis. Milk was pasteurized at 85 °C for 15 s.

Six bags of freeze-dried jenny milk powder (**FM**, vacuum degassed), produced from the same tracked batch after pasteurization, were taken randomly. Milk powder samples were reconstituted with 1:11 (weight/volume) double distilled sterile water, and 200 mL of each reconstituted FM sample was divided into 5 aliquots for analyses. After pasteurization, the milk was lyophilized at 40 to 50 °C under −0.05 MPa pressure until the water content was <30%. Then, the condensed milk was dehydrated under vacuum at −40 to −70 °C and 150 Pa pressure for approximately 48 h until the water content was ≤3%. The RM samples were from individual jenny, whereas DM/ PM/ FM samples were taken from pooled milk derived from the same production batch.

### 2.3 Sample analyses

#### 2.3.1 Milk proximate components and pH

Milk fat, non-fat solids, lactose, protein, ash, density were measured immediately after collection in RM (*n* = 7), DM (*n* = 8), PM (*n* = 6), and FM (n = 6) samples using a calibrated milk component analyzer (UL40BC, Hangzhou Ultrasun Technologies CO. Ltd., Hangzhou, China), and pH was measured with a pH meter (FE28, Mettler Toledo, Shanghai, China). Each sample was measured in duplicate, and the average value was used for analyses.

#### 2.3.2 Untargeted metabolomics

Samples of RM (n = 7), DM (n = 8), PM (n = 6), and FM (n = 6) were thawed on ice, and vortexed for 10 s. Fifty μL of milk were added to 300 μL of extraction solution containing 20% acetonitrile-methanol internal standard, vortexed for 3 min, and centrifuged at 15,200×*g* for 10 min at 4 °C. Then, 200 μL of the supernatant were kept at −20 °C for 30 min, centrifuged at 15,200×*g* for 3 min at 4 °C, and 180 μL of the supernatant were taken for analysis. Ultra-high performance liquid chromatography-tandem time-of-flight mass spectrometry (UHPLC-Q-TOF MS, LC-30A, Shimadzu, Kyoto, Japan) with AB TripleTOF 6600 mass spectrometer (SCIEX, Foster City, CA, USA) were used to detect metabolites. The following conditions were used - chromatographic column: Waters ACQUITY Premier HSS T3 Column (1.8 μm × 2.1 mm × 100 mm, Milford, MA, USA); mobile phase A: 0.1% formic acid in water; mobile phase B: 0.1% formic acid in acetonitrile. Instrument column temperature: 40 °C; flow rate: 0.4 mL/min; injection volume: 4 μL. Details of measurements and data analyses are presented in Supplement file 1.

#### 2.3.3 Trace elements

Samples of RM (*n* = 4), DM (*n* = 3), PM (n = 3), and FM (n = 3) were selected randomly, thawed and vortexed for 1 min. Essential and probably essential elements (Co, Cu, I, Fe, Mn, Mo, Se, Zn, Ni and Cr), and potentially toxic elements (Pb, Cd, Hg, As and Sn) were determined using inductively coupled plasma mass spectrometry (ICP-MS) (Agilent 7800, Agilent Technologies, Inc., Santa Clara, CA, USA). Detailed methods are presented in Supplement file 1.

#### 2.3.4 Free short- and medium-chain fatty acids (FSMCFA)

Samples of RM (*n* = 4), DM (*n* = 3), PM (n = 3), and FM (n = 3) were selected randomly, thawed and vortexed for 1 min. Then 50 μL were added to 200 μL of phosphoric acid solution (0.5%, *v*/v), vortexed for 10 min, sonicated at 4 °C for 5 min, and centrifuged at 15,200×*g* at 4 °C for 10 min. Then, 100 μL of the supernatant were added to 250 μL of MTBE extract containing an internal standard, vortexed for 3 min, sonicated at 4 °C for 5 min, centrifuged at 15,200×*g* at 4 °C for 10 min, and 100 μL of the supernatant were used for determination of FFA concentrations using the Agilent 8890-7000D GC–MS/MS platform. The internal standard method was applied to quantify 17 SCFA and MCFA (acetic, propionic, isobutyric, butyric, 2-methylbutyric, isovaleric, valeric, isohexanoic, hexanoic, isoheptanoic, heptanoic, 2-ethylhexanoic, caprylic, nonanoic, 3,5,5-trimethylhexanoic, isodecanoic, and decanoic acids). Detailed methods are presented in Supplement file 1.

#### 2.3.5 Entire medium and long-chain fatty acids (MLCFA)

Samples of RM (n = 4), DM (n = 3), PM (n = 3), and FM (n = 3) were selected randomly, thawed and vortexed for 10 s. A 50 μL sample was added to 150 μL of methanol solution, 200 μL of methyl tert-butyl ether solution, and 50 μL of 36% phosphoric acid solution as extraction solutions. The mixture was vortexed for 3 min and then was centrifuged at 15,200×*g* at 4 °C for 5 min. Then, 200 μL of the supernatant were dried using a nitrogen blower, added to 300 μL of 15% boron trifluoride methanol solution, vortexed for 3 min, and kept at 60 °C in an oven for 30 min. The sample was cooled to room temperature and added to 500 μL of n-hexane solution and 200 μL of saturated sodium chloride solution, vortexed for 3 min, and centrifuged at 15,200×*g* at 4 °C for 2 min. Then. 100 μL of the upper layer solution was pipetted for analysis. The sample derivants were analyzed using gas chromatography (Agilent 7890B, Santa Clara, CA, USA) coupled to triple quadrupole mass spectrometry (Agilent 7000D). The external standard method was used for quantitative determination of 48 MLCFA. Detailed methods are presented in Supplement file 1.

### 2.4 Data analyses

Metabolome data preprocessing, orthogonal partial least-squares discriminant analysis (OPLS-DA), and Kyoto encyclopedia of genes and genomes analysis (KEGG) followed Zhang et al. (2026). Differential metabolite screening combined multivariate and univariate criteria. The variable importance in projection (VIP) value was obtained from the OPLS-DA, and the univariate *P*-value was determined for each metabolite. Metabolites with VIP > 1 and a nominal *P* < 0.05 were defined as candidate differential metabolites.

After testing for normality and homogeneity, milk proximate components, pH, trace elements and FA were analyzed using a one-way ANOVA (SPSS 19.0, IBM SPSS Inc. New York, NY, USA), with milk type (RM, DM, PM, and FM) as a fixed factor. The post hoc Duncan's multiple range test compared treatments for homogeneous variables while the Welch ANOVA and the Games-Howell test were used for non-homogeneous variables. If not distributed normally, the data were analyzed by the non-parametric Kruskal-Wallis's test using GraphPad Prism 8 (Boston, MA, USA), and separated by Dunn's test. Data are presented as means ± SEM, and statistical significance was accepted at *P* ˂ 0.05.

## 3 Results

### 3.1 Milk proximate components and pH

Protein and ash contents were lower (*P* < 0.05) in RM than in DM, PM, and FM (Table 1); lactose content was lower (*P* < 0.05) in RM than in DM and PM; and density was lower (*P* < 0.05) in RM than in DM. Protein content was lower (*P* < 0.05) in DM and PM than in FM.

**Table 1:** Proximate components in different types of jenny milk.

Item	Milk type^1^				SEM	*P*-value
	RM (n = 7)	DM (n = 8)	PM (n = 6)	FM (n = 6)		
Fat (g/kg)	8.8	5.2	6.4	4.3	1.59	0.480
Non-fat solid (g/kg)	79.9	84.2	84.1	84.1	0.63	0.204
Lactose (g/kg)	60.5^b^	62.9^a^	62.8^a^	61.4^ab^	0.35	0.010
Protein (g/kg)	13.5^c^	15.1^b^	15.1^b^	16.5^a^	0.28	0.001
Ash (g/kg)	5.7^b^	6.0^a^	6.0^a^	6.1^a^	0.05	0.014
Density (g/cm^3^)	1.027^b^	1.030^a^	1.029^ab^	1.030^ab^	0.0003	0.002
pH	7.02	7.02	6.80	6.86	0.058	0.462

1 RM = Fresh raw milk from individual jennies; DM = Raw pooled milk from depository; PM = Pasteurized pooled milk; FM = Freeze-dried pooled milk.

### 3.2 Metabolites in milk

#### 3.2.1 Data quality evaluation

From the total ion current (TIC) chromatograms, Pearson correlation analyses, internal standard stability, and coefficient of variation analysis (Supplement file 1) from mass spectrometry detection and analysis of quality control (QC) samples, it emerged that the results of metabolite extraction and detection were highly repeatable and reliable, and that the experimental data were stable and of high-quality.

#### 3.2.2 Analysis of differential metabolites

**Fig. 1:**
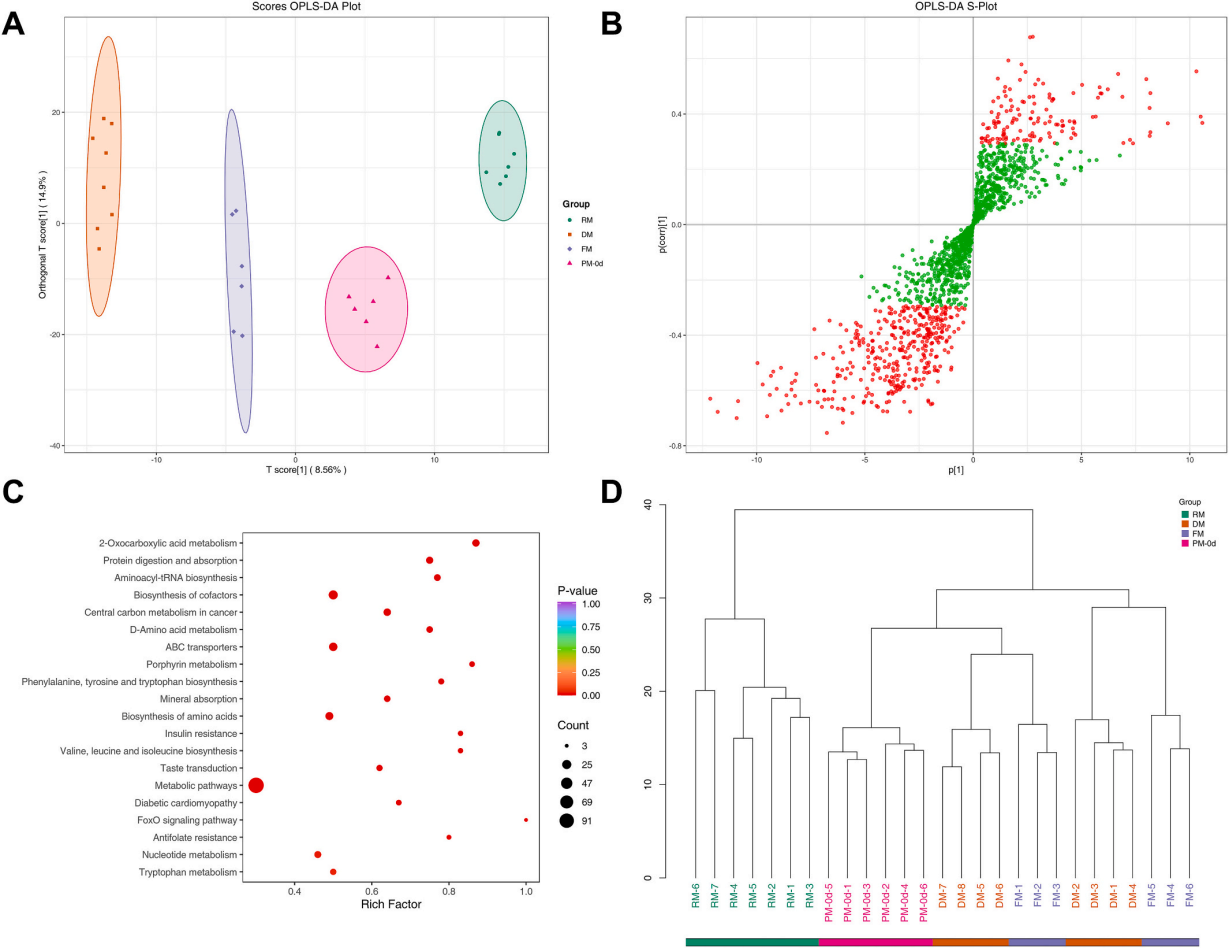
Differential metabolites and Kyoto encyclopedia of genes and genomes (KEGG) analysis among different jenny milk types. A. OPLS-DA score Plot: x-axis represents the predictive principal components; y-axis represents the orthogonal principal components; the percentage value of axis represents the explanatory rate of that component to the dataset. B. OPLS-DA S-Plot: x-axis represents the covariance between principal components and metabolites, and y-axis represents the correlation coefficient between principal components and metabolites. Metabolites which are closer to the upper right corner and lower left corner indicate more significant differences. Red dots indicate that the variable importance in projection (VIP) values of these metabolites is greater than 1, and green dots indicate that the VIP values of these metabolites are less than or equal to 1. C. KEGG enrichment analysis: x-axis represents the rich factor corresponding to each pathway, and y-axis is the pathway name (sorted by *P*-value). The color of the dots reflects the size of the *P*-value, the size of the dots represents the number of differential metabolites enriched. D. Hierarchical clustering dendrogram: each branch in the plot represents a sample.

A total of 1505 metabolites were detected and putatively annotated in the 4 milk types, including 819 in the positive ion mode and 686 in the negative ion mode. There were 354 candidate differential metabolites (Supplemental Table 1, Fig. 1A and B), including 60 amino acids and their metabolites, 56 benzenes and their derivatives, 21 alcohols and amines, 43 glycerophospholipids, glycerides, fatty acyls and sphingolipids, 21 nucleotides and their metabolites, 40 heterocyclic compounds, 43 organic acids and their derivatives, 22 carbohydrates and their metabolites, and the rest were terpenoids, alkaloids, tryptamines, choline, pigments, and drugs. KEGG enrichment analysis indicated that the candidate differential metabolites were associated mainly with broad metabolic categories, including amino acid, nucleotide, carbohydrate, and lipid-related metabolism and, that overall, the metabolites changed patterns (Fig. 1C). Furthermore, the metabolite profiles of RM differed from DM, FM, and PM, which did not differ from each other (Fig. 1D).

**Fig. 2:**
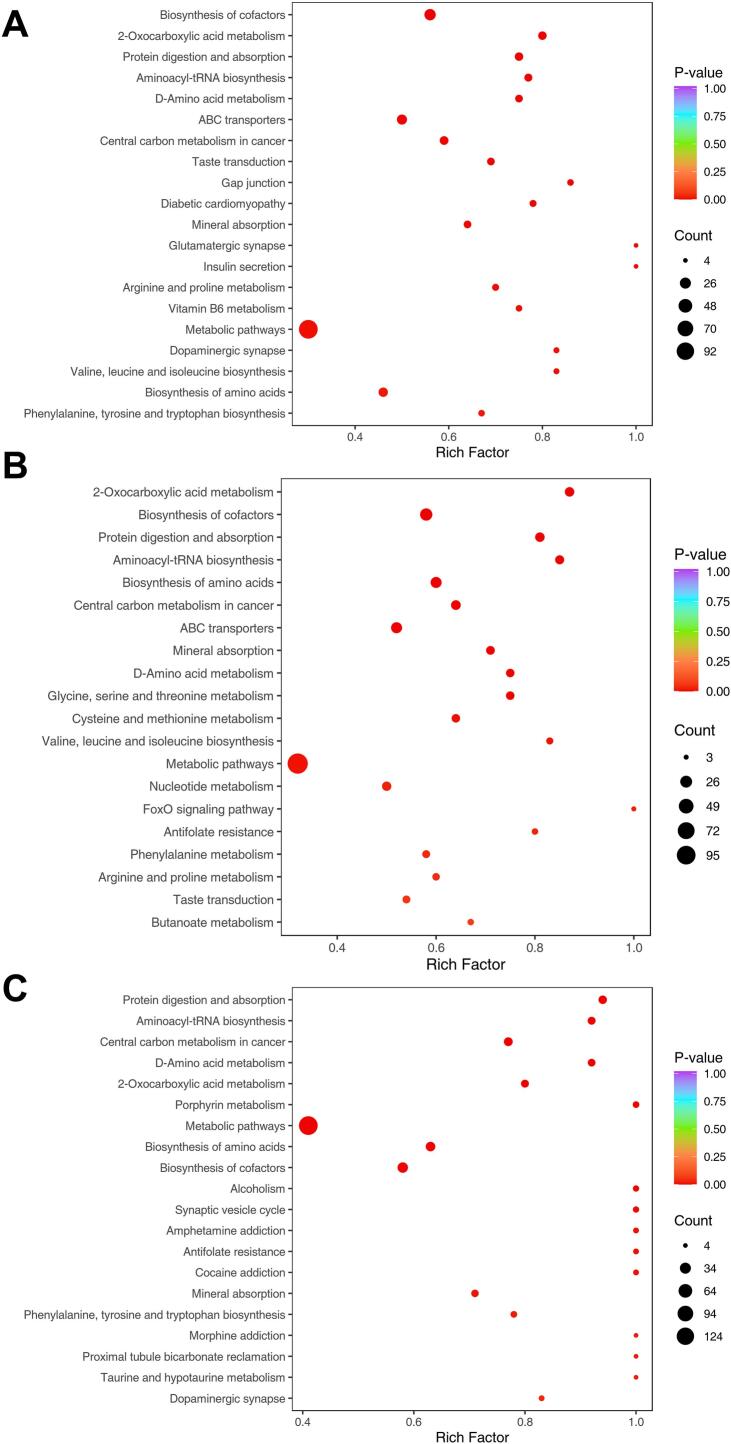
Kyoto encyclopedia of genes and genomes (KEGG) analysis of differential metabolites of raw pooled milk from depository (DM), pasteurized pooled milk (PM) and freeze-dried pooled milk (FM) compared with fresh raw milk from individual jennies (RM). A. Comparison between DM and RM; B. Comparison between PM and RM; C. Comparison between FM and RM.

**Fig. 3:**
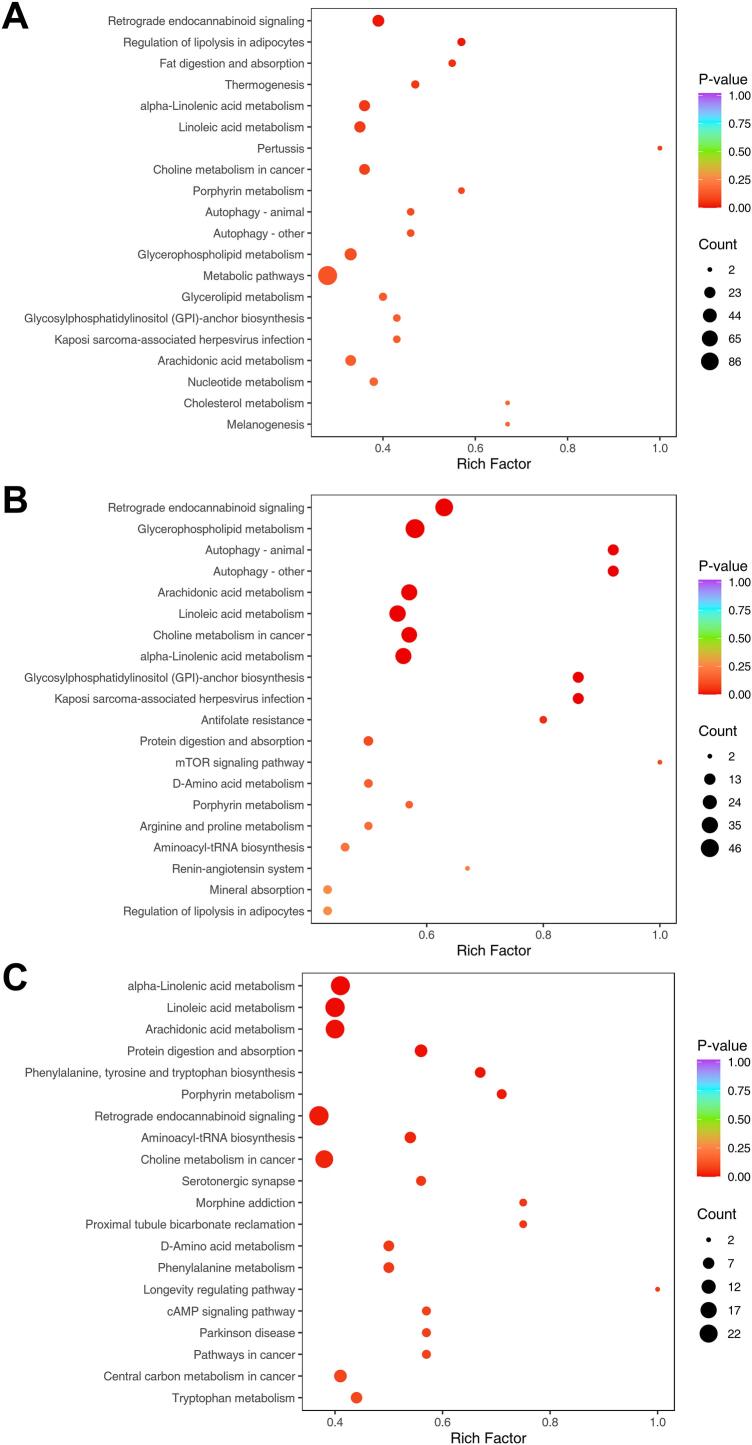
Kyoto encyclopedia of genes and genomes (KEGG) analysis of differential metabolites among freeze-dried pooled milk (FM), pasteurized pooled milk (PM) and raw pooled milk from depository (DM). A. Comparison between PM and DM; B. Comparison between FM and DM; C. Comparison between FM and PM.

Compared with RM, the candidate differential metabolites of DM, FM, and PM were 326 (74 down-regulated and 252 up-regulated), 425 (135 down-regulated and 290 up-regulated), and 556 (229 down-regulated and 327 up-regulated) respectively, which were also related mainly to protein, amino acid, nucleotide, and carbon metabolic pathways (Fig. 2A, B, and C). Compared with DM, the candidate differential metabolites of PM and FM were 512 (262 down-regulated and 250 up-regulated) and 399 (177 down-regulated and 222 up-regulated), respectively. The differential pathways in these two milk types were involved mainly in lipid metabolism, including alpha-linolenic acid metabolism, linoleic acid metabolism, arachidonic acid metabolism, glycerophospholipid metabolism, and glycerolipid metabolism. The remaining metabolites included retrograde endocannabinoid signaling and choline metabolism in cancer (Fig. 3A and B).

Between FM and PM, there were 390 candidate differential metabolites (159 down-regulated and 231 up-regulated). In addition to retrograde endocannabinoid signaling, choline metabolism in cancer and metabolic pathways, the remaining pathways were mostly lipid-related, such as alpha-linolenic acid metabolism, linoleic acid metabolism, and arachidonic acid metabolism (Fig. 3C).

### 3.3 Trace elements

In all samples, 15 elements, namely, Co, Cu, Mn, Fe, Se, Zn, I, Mo, Pb, Cd, Hg, As, Sn, Ni, and Cr, were detected (Table 2). The concentrations of Co and Mn in FM were higher (*P* < 0.05) than in RM and PM; of Zn in FM was higher (*P* = 0.002) than in RM and DM; of I in PM was higher (*P* = 0.031) than in RM and DM; of Ni in FM was higher (*P* = 0.001) than in DM; of Cr in FM was higher (*P* < 0.05) than in PM; and of As in FM was higher (*P* < 0.05) than in RM, DM and PM.

**Table 2:** Concentrations of essential and toxic trace elements in different types of jenny milk.

Concentration (μg/L)	Milk type^1^				SEM	*P*-value
	RM (*n* = 4)	DM (*n* = 3)	PM (n = 3)	FM (n = 3)		
Cobalt (Co)	0.88^b^	2.48^ab^	1.07^b^	9.32^a^	1.111	0.023
Copper (Cu)	82.6	93.3	107.2	96.8	4.57	0.326
Manganese (Mn)	13.7^b^	18.8^ab^	13.7^b^	24.8^a^	1.71	0.032
Iron (Fe)	2478	2031	223	767	516.1	0.224
Selenium (Se)	14.7	14.0	10.1	17.1	1.56	0.260
Zinc (Zn)	1572^b^	1931^b^	2160^ab^	2661^a^	130.4	0.002
Iodine (I)	10.0^b^	10.3^b^	52.2^a^	26.8^ab^	6.15	0.031
Molybdenum (Mo)	13.8	15.1	11.8	13.1	1.00	0.769
Nickel (Ni)	12.1^ab^	4.49^b^	7.41^ab^	32.4^a^	3.388	0.001
Chromium (Cr)	12.1^ab^	7.07^ab^	5.88^b^	31.72^a^	3.361	0.038
Lead (Pb)	10.9	16.4	14.5	10.0	1.64	0.171
Cadmium (Cd)	0.30	0.19	0.38	0.20	0.035	0.184
Mercury (Hg)	0.30	0.20	0.46	0.30	0.055	0.453
Arsenic (As)	0.96^b^	0.91^b^	1.07^b^	2.12^a^	0.171	0.006
Tin (Sn)	3.20	3.29	7.25	9.77	1.216	0.168

1 RM = Fresh raw milk from individual jennies; DM = Raw pooled milk from depository; PM = Pasteurized pooled milk; FM = Freeze-dried pooled milk.

### 3.4 Fatty acids

#### 3.4.1 Free short- and medium-chain fatty acids (FSMCFA)

Among FSMCFA, acetic acid was detected in some DM and RM samples, and nonanoic acid was detected in some DM, RM, and PM samples. Decanoic, caprylic, and 2-methylbutyric acids were the most abundant FSMCFA in each group (Table 3).

**Table 3:** Concentrations of free short-chain and medium-chain fatty acids among different types of jenny milk.

Concentration (μg/mL)	Milk type^1^				SEM	*P*-value
	RM (n = 4)	DM (n = 3)	PM (n = 3)	FM (n = 3)		
Acetic acid	–	–	1.18	1.74	0.161	0.167
Propionic acid	0.27^b^	0.32^ab^	0.39^a^	0.37^a^	0.023	0.012
Isobutyric acid	0.04^c^	0.05^bc^	0.07^a^	0.06^ab^	0.005	0.019
Butyric acid	0.50^b^	0.58^b^	0.74^b^	2.30^a^	0.132	0.001
2-Methylbutyric acid	4.22	3.54	3.26	3.81	0.322	0.104
Isovaleric acid	0.03^d^	0.04^c^	0.06^b^	0.07^a^	0.003	<0.001
Valeric acid	0.02	0.02	0.02	0.02	0.004	0.623
Hexanoic acid	0.74^b^	0.39^b^	0.54^b^	1.75^a^	0.257	0.005
Heptanoic acid	0.16	0.11	0.12	0.13	0.013	0.122
Octanoic acid	8.39^b^	4.80^b^	6.59^b^	15.3^a^	2.276	<0.001
Decanoic acid	11.4^ab^	8.15^b^	10.4^b^	23.5^a^	2.59	<0.001
Total	26.3^ab^	18.2^b^	23.4^b^	49.1^a^	3.92	<0.001

1 RM = Fresh raw milk from individual jennies; DM = Raw pooled milk from depository; PM = Pasteurized pooled milk; FM = Freeze-dried pooled milk.

**Fig. 4:**
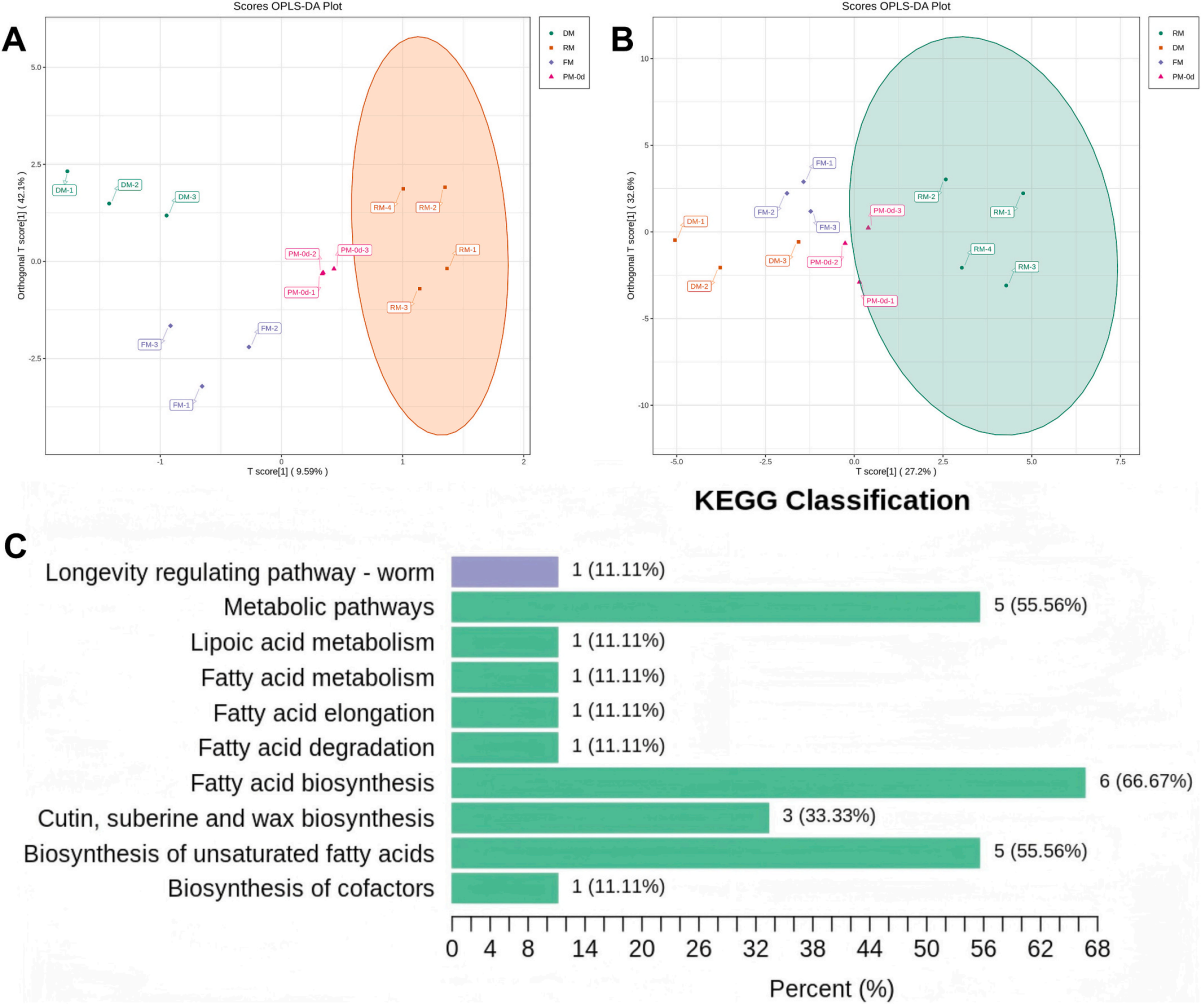
Fatty acid composition among different jenny milk types. A. Orthogonal partial least-squares discriminant analysis (OPLS-DA) plot of free short- and medium-chain fatty acids; B. OPLS-DA plot of entire medium- and long-chain fatty acids; C. Kyoto encyclopedia of genes and genomes (KEGG) enrichment analysis of medium- and long-chain fatty acids.

The concentrations of propionic, isobutyric, and isovaleric acids were lower (*P* < 0.05) in RM than in PM and FM. The concentrations of butyric, isovaleric, hexanoic, and caprylic acids were higher (*P* < 0.05) in FM than in the other three milk types, and the concentrations of decanoic acid and total FSMCFA were higher (*P* < 0.05) in FM than in DM and PM. Based on the OPLS-DA model (Fig. 4A), the FSMCFA profile of RM differed from the other milk types.

#### 3.4.2 Entire medium and long-chain fatty acids (MLCFA)

Of the MLCFA, the concentration of palmitic acid was highest, followed by oleic, linoleic, stearic, lauric, and α-linolenic acids (Table 4, Table 5). The odd‑carbon FA accounted for only 0.71% (0.63–0.85%) and of the UFA accounted for 43.5% (39.7–47.2%), while the ratio of n-3 FA: n-6 FA ranged between 1:1.3 and 1:3.7.

**Table 4:** Concentrations of entire middle- and long-chain fatty acids among different types of jenny milk.

Fatty acids^2^ (μg/mL)	Milk type^1^				SEM	*P*-value
	RM (n = 4)	DM (n = 3)	PM (n = 3)	FM (n = 3)		
Hexanoic acid	18.6^a^	8.44^b^	9.34^ab^	10.4^ab^	1.47	0.004
Octanoic acid	174.9	80.8	83.4	79.9	14.44	0.096
Nonanoic acid	2.16	1.04	1.11	1.03	0.191	0.095
Decanoic acid	236^a^	129^ab^	139^ab^	117^b^	17.4	0.043
Undecanoic acid	1.89^a^	0.68^ab^	0.78^ab^	0.66^b^	0.321	0.036
Lauric acid	218^a^	127^ab^	133^ab^	109^b^	22.9	0.040
Myristic acid	158.4	98.4	108.4	100.2	13.34	0.051
Myristoleic acid	9.23	7.04	7.62	7.72	0.676	0.147
Pentadecanoic acid	7.38	5.23	6.04	7.40	0.66	0.103
Palmitic acid	562	481	537	569	22.3	0.071
cis-9-Palmitoleic acid	88.2^b^	71.7^b^	84.8^b^	128^a^	9.03	0.009
Hexadecanedioic acid	0.87	0.86	0.87	0.87	0.014	0.826
Heptadecanoic acid	6.63^a^	4.32^c^	5.26^bc^	5.55^ab^	0.377	0.008
Stearic acid	204^b^	219^ab^	246^a^	224^ab^	8.90	0.040
cis-9-Octadecenoic acid/ Oleic acid	441^a^	286^b^	347^b^	445^a^	29.6	0.009
trans-9-Octadecenoic acid	20.2^b^	16.1^b^	19.6^b^	26.2^a^	1.87	0.025
Linoleic acid	331^ab^	228^b^	278^ab^	363^a^	31.82	0.020
Linolelaidic acid	5.86	5.34	5.05	5.69	0.435	0.508
α-Linolenic acid	122.9	161.8	114.4	89.7	21.48	0.123
γ-Linolenic acid	1.25	1.04	1.22	1.36	0.092	0.172
Nonadecanoic acid	0.50^a^	0.38^b^	0.37^b^	0.45^a^	0.02	0.002
Arachidic acid	2.82	2.42	2.77	2.73	0.125	0.155
cis-11-Eicosenoic acid	7.86^a^	4.46^c^	5.48^bc^	6.67^ab^	0.593	0.010
cis-11,14-Eicosadienoic acid	6.88^ab^	5.29^b^	6.10^ab^	7.30^a^	0.507	0.028
cis-11,14,17-Eicosatrienoic acid	3.65	4.12	3.54	3.20	0.388	0.453
cis-8,11,14-Eicosatrienoic acid	1.64^ab^	1.36^b^	1.48^ab^	1.71^a^	0.103	0.037
cis-5,8,11,14-Eicosatetraenoic acid	1.99	1.51	1.84	2.85	0.314	0.072
Heneicosanoic acid	0.90	0.88	0.90	0.90	0.006	0.052
Behenic acid	0.78	0.51	0.62	0.63	0.073	0.113
Erucic acid	3.06	2.45	5.43	3.00	1.476	0.680
cis-13,16-Docosadienoic acid	0.69	0.67	0.69	0.82	0.068	0.391
cis-4,7,10,13,16,19-Docosahexaenoic acid	6.92	6.82	6.82	6.88	0.038	0.205
Lignoceric acid	0.31	0.23	0.23	0.30	0.042	0.272
MCFA	651	348	366	318	49.2	0.071
LCFA	1615	2010	1796	1995	62.7	0.062
SFA	1603	1167	1281	1237	62.2	0.080
UFA	1043	797	882	1092	45.1	0.051
MUFA	561^ab^	382^c^	463^bc^	610^a^	29.7	0.010
n-3 PUFA	134	173	125	100	11.7	0.125
n-6 PUFA	341^ab^	237^b^	288^ab^	375^a^	20.0	0.019
n-9 UFA	552^ab^	376^c^	457^bc^	602^a^	29.3	0.010
PUFA	482	416	419	483	22.8	0.133
Total	2647	1964	2164	2329	98.3	0.056

1 RM = Fresh raw milk from individual jennies; DM = Raw pooled milk from depository; PM = Pasteurized pooled milk; FM = Freeze-dried pooled milk.

2 MCFA: Medium chain fatty acid (C6-C12 FA); LCFA: Long chain fatty acid (C14-C22 FA); SFA: Saturated fatty acid; UFA: Unsaturated fatty acid; MUFA: Monounsaturated fatty acid; n-3 PUFA: included α-linolenic acid, cis-11,14,17-eicosatrienoic acid and cis-4,7,10,13,16,19-docosahexaenoic acid; n-6 PUFA: included linoleic acid, trans-linoleic acid, γ-linolenic acid, cis-8,11,14-eicosatrienoic acid and arachidonic acid; n-9 UFA: included cis-9-palmitoleic acid, oleic acid, elaidic acid and erucic acid; PUFA: polyunsaturated fatty acid.

**Table 5:** Percentages of entire middle- and long-chain fatty acids in different types of jenny milk.

Fatty acids^2^ (%)		Milk type^1^			SEM	*P*-value
	RM (n = 4)	DM (n = 3)	PM (n = 3)	FM (n = 3)		
Hexanoic acid	0.69	0.43	0.43	0.45	0.138	0.055
Octanoic acid	6.53^a^	4.12^b^	3.83^b^	3.43^b^	1.483	0.001
Nonanoic acid	0.08^a^	0.05^b^	0.05^b^	0.04^b^	0.020	0.040
Decanoic acid	8.78^a^	6.62^b^	6.40^b^	5.03^b^	1.658	0.003
Undecanoic acid	0.07^a^	0.03^ab^	0.04^ab^	0.03^b^	0.022	<0.001
Lauric acid	8.12^a^	6.50^ab^	6.13^ab^	4.67^b^	1.618	0.015
Myristic acid	5.93^a^	5.02^b^	5.01^b^	4.30^b^	0.728	0.005
Myristoleic acid	0.35	0.36	0.35	0.33	0.031	0.785
Pentadecanoic acid	0.28	0.27	0.28	0.32	0.036	0.326
Palmitic acid	21.5	24.5	25.0	24.4	2.23	0.433
cis-9-Palmitoleic acid	3.35^b^	3.63^b^	3.90^b^	5.48^a^	0.976	0.004
Heptadecanoic acid	0.25	0.22	0.24	0.24	0.021	0.074
Stearic acid	7.87^b^	11.18^a^	11.43^a^	9.62^ab^	1.855	0.011
cis-9-Octadecenoic acid/Oleic acid	16.9	14.5	16.0	19.1	2.37	0.087
trans-9-Octadecenoic acid	0.77^b^	0.81^b^	0.91^ab^	1.13^a^	0.185	0.043
Linoleic acid	12.4^b^	11.6^b^	12.8^b^	15.6^a^	1.83	0.009
Linolelaidic acid	0.22^b^	0.27 ^a^	0.23^ab^	0.24^ab^	0.024	0.023
α-Linolenic acid	4.51^ab^	8.31^a^	5.27^ab^	3.85^b^	2.119	0.036
γ-Linolenic acid	0.05^b^	0.05^ab^	0.06^a^	0.06^a^	0.006	0.045
Nonadecanoic acid	0.02	0.02	0.02	0.02	0.001	0.161
Arachidic acid	0.11	0.12	0.13	0.12	0.012	0.114
cis-11-Eicosenoic acid	0.30	0.23	0.25	0.29	0.043	0.087
cis-11,14-Eicosadienoic acid	0.26^b^	0.27^b^	0.28^b^	0.31^a^	0.024	0.002
cis-11,14,17-Eicosatrienoic acid	0.14^b^	0.21^a^	0.16^b^	0.14^b^	0.037	0.025
cis-8,11,14-Eicosatrienoic acid	0.06^b^	0.07^ab^	0.07^ab^	0.07^a^	0.006	0.035
cis-5,8,11,14-Eicosatetraenoic acid	0.07^b^	0.08^b^	0.09^b^	0.12^a^	0.026	0.038
Heneicosanoic acid	0.03	0.04	0.04	0.04	0.005	0.084
Behenic acid	0.03	0.03	0.03	0.03	0.003	0.555
Erucic acid	0.11	0.13	0.24	0.13	0.103	0.796
cis-13,16-Docosadienoic acid	0.03	0.03	0.03	0.04	0.006	0.136
cis-4,7,10,13,16,19-Docosahexaenoic acid	0.27	0.35	0.32	0.30	0.041	0.067
Lignoceric acid	0.01	0.01	0.01	0.01	0.002	0.703
MCFA	24.3^a^	17.8^b^	16.9^b^	13.7^b^	4.81	0.003
LCFA	75.7^b^	82.2^a^	83.1^a^	86.3^a^	4.81	0.003
UFA	39.7^b^	40.8^b^	41.0^b^	47.2^a^	3.30	0.001
SFA	60.3^a^	59.2^a^	59.0^a^	52.8^b^	3.30	0.001
n-3FA	4.91^b^	8.87^a^	5.75^b^	4.29^b^	2.173	0.008
n-6FA	12.8^b^	12.0^b^	13.3^b^	16.1^a^	1.85	0.008
n-9FA	21.1^b^	19.1^b^	21.1^b^	25.9^a^	3.28	0.040
PUFA	18.0	21.2	19.4	20.7	2.12	0.168
MUFA	21.7^b^	19.6^b^	21.7^b^	26.5^a^	3.31	0.042

1 RM = Fresh raw milk from individual jennies; DM = Raw pooled milk from depository; PM = Pasteurized pooled milk; FM = Freeze-dried pooled milk.

2 MCFA: Medium chain fatty acid (C6-C12 FA); LCFA: Long chain fatty acid (C14-C22 FA); SFA: Saturated fatty acid; UFA: Unsaturated fatty acid; MUFA: Monounsaturated fatty acid; n-3 PUFA: included α-linolenic acid, cis-11,14,17-eicosatrienoic acid and cis-4,7,10,13,16,19-docosahexaenoic acid; n-6 PUFA: included linoleic acid, trans-linoleic acid, γ-linolenic acid, cis-8,11,14-eicosatrienoic acid and arachidonic acid; n-9 UFA: included cis-9-palmitoleic acid, oleic acid, elaidic acid and erucic acid; PUFA: polyunsaturated fatty acid.

For FA concentrations (Table 4), hexanoic acid was higher (*P* = 0.004) in RM than in DM, and decanoic acid was higher (*P* = 0.043) in RM than in FM. Lauric acid was higher (*P* = 0.020) in RM than in FM, and heptadecanoic acid (*P* = 0.008) and cis-11-eicosenoic acid (*P* = 0.010) were higher in RM than in DM and PM. Stearic acid concentration was higher (*P* = 0.040) in PM than in RM; cis-9-palmitoleic acid and elaidic acid were higher (*P* < 0.05) in FM than in the other milk types; oleic acid and nonadecanoic acid were higher (*P* < 0.05) in FM and RM than in DM and PM; linoleic acid, cis-11,14-eicosadienoic acid, and cis-8,11,14-eicosatrienoic acid were higher (*P* < 0.05) in FM than in DM; MUFA and n-6 PUFA were higher (*P* < 0.05) in FM than in DM and PM; and, the n-6 PUFA was higher (*P* < 0.05) in FM than in DM.

For FA percentage (Table 5), caprylic, nonanoic, decanoic, undecanoic, myristic, and MCFAs were higher (*P* < 0.05) in RM than in the other milk types; lauric acid was higher (*P* = 0.015) in RM than in FM; cis-9-palmitoleic acid, linoleic acid, cis-11,14-eicosadienoic acid, arachidonic acid, UFA, n-6FA, n-9FA, and MUFA were higher (*P* < 0.05) in FM than in the other three milk types; linolelaidic acid was higher (*P* = 0.023) in DM than in RM, and α-linolenic acid was higher (*P* = 0.036) in DM than in FM; cis-11,14,17-eicosatrienoic acid, and n-3 PUFA were higher (*P* < 0.05) in DM than in the other three milk types, and stearic acid were higher (*P* = 0.011) in DM and PM than in RM and FM. The γ-linolenic acid, cis-8,11,14-eicosatrienoic acid, and LCFA were lowest (*P* < 0.05) in RM among the four milk types.

From the OPLS-DA and KEGG diagrams (Fig. 4B, and C), the FA profile of RM differed from the other three milk types, and the differential FA was characterized mainly by UFA.

### 3.5 Correlation analyses

**Fig. 5:**
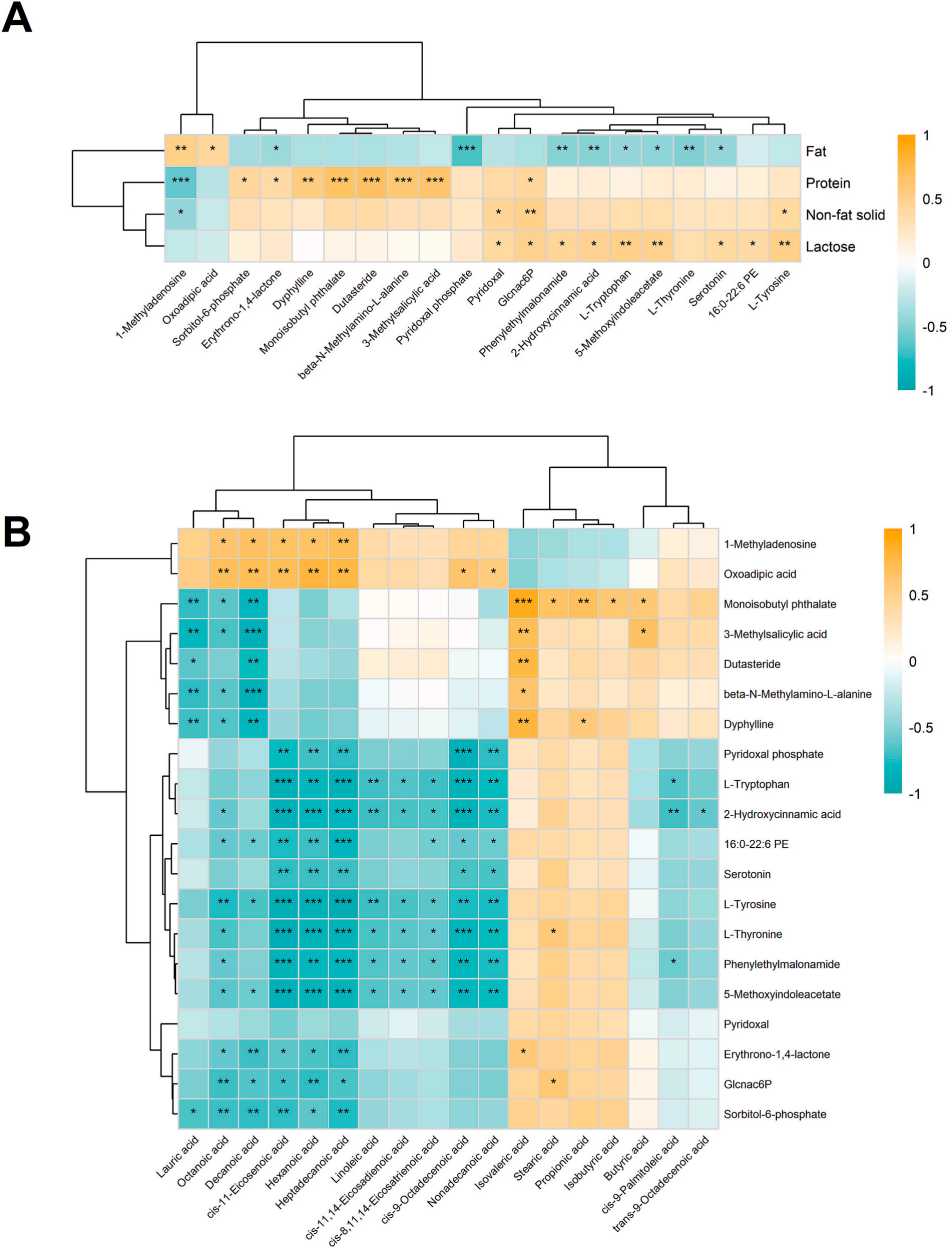
Spearman correlation heatmap between proximate components and the top 20 candidate differential metabolites. A. Between proximate components and the top 20 candidate differential metabolites; B. Between differential fatty acids and the top 20 candidate differential metabolites.

Correlations between milk proximate components and candidate differential metabolites are presented in Fig. 5A. Milk protein and lactose were correlated positively with the candidate metabolites associated with amino acid-, carbohydrate-, and aromatic compound-related metabolites. Milk fat was correlated negatively with most of these metabolites, whereas non-fat solids were correlated positively with some of these metabolites.

Correlations between differential FA and candidate differential metabolites are presented in Fig. 5B. Most differential MCFA and LCFA were correlated negatively with differential metabolites, except for 1-methyladenosine and oxoadipic acid. Conversely, short-chain and branched-chain FA were correlated positively with most of these amino acid-, aromatic compound-, carbohydrate-, and phospholipid-related candidate metabolites.

In correlations between milk ash and trace elements, only Pb was correlated positively with ash content (R^2^ = 0.740, *P* = 0.006), while the other trace elements were not (*P* > 0.05, data not presented).

## 4 Discussion

Jenny milk is very nutritious, but raw milk ferments and spoils rapidly at room temperature and is intolerant of low and high temperatures (Luo et al., 2019; Miao et al., 2020; Tedeschi et al., 2023). Currently, pasteurization and lyophilization are effective methods for preserving the components of jenny milk (Seyiti & Kelimu, 2021). The aim of the present study was to determine the compositional changes in jenny milk among different processing stages, including raw milk from individual jennies, pooled raw milk after cold storage, pasteurized milk and freeze-dried milk powder. This study was conducted in a commercial farm-scale dairy and, therefore, the results are relevant for an industrial production enterprise. All samples were collected from the same farm within the same production batch, which reduced potential variation related to season, feeding management, and production conditions. Although sample sizes differed among milk types and some samples were small, the present study integrated multi-dimensional profiling on the physicochemical composition, metabolite profiles, trace elements, and FA profiles of jenny milk from raw fresh milk to commercial processed products on the same batch of milk. The results have important implications for jenny milk producers in improving processing parameters.

### 4.1 Proximate components and pH

The concentrations of protein, lactose, ash and density in RM were lower than the other milk types, (DM, PM and FM), which did not differ among them except for protein. The RM was derived from each jenny at mid-lactation stage, and at this stage the milk solids starting decreasing (Fantuz et al., 2025). However, the components and pH of the four milk types in the present study were similar to those reported previously (Garhwal et al., 2023; Živkov Baloš et al., 2023).

### 4.2 Untargeted metabolome

Untargeted metabolomics analysis aims to understand the overall difference and dynamic changes of metabolites (Zhu et al., 2020). In the present study, compared with RM, the differential metabolite profiles and pathways of the other three milk types were similar, mainly involved with non-lipid components such as proteins, amino acids, nucleotides, cofactors, and carbohydrates. This indicated that the non-lipid composition of jenny milk was altered after milking and during cryopreservation. Raw milk contains various endogenous and microbial enzymes, including alkaline phosphatase, proteases, lactoperoxidase, and catalase, which may remain active before complete cooling or heating (Alan & Patrick, 2006; Deeth, 2021; Eyassu et al., 2005). These enzymes led to the degradation and transformation of milk components, which could explain the metabolite differences post-milking. Thus, shortening the time between milking and cooling is critical to preserve the non-lipid components. Most candidate differential metabolites were correlated positively with the non-fat milk fraction, but negatively with milk fat, which supported our suggestion. Next, measuring enzyme activities would be beneficial in understanding the mechanisms.

Compared with DM, the differential pathways in PM and FM included mainly alpha-linolenic acid, linoleic acid, glycerophospholipid and arachidonic acid metabolism because heat treatment promoted the hydrolysis and oxidation of milk lipids (Zhang et al., 2022), with UFA being more susceptible. In addition, differential metabolites and pathways related to proteins, amino acids, and cofactors were also influenced, which could be due to heat causing structural and chemical denaturation, aggregation, Maillard reaction, and enzymatic modifications in milk proteins and lactose (Lu et al., 2023; Rabbani et al., 2025; Yan, Zhang, et al., 2022), thus altering protein and amino acid components. Therefore, more attention should be focused on changes in lipids during thermal processing of jenny milk to improve the preservation of lipid components in the milk.

Between FM and PM, the main differential pathways were the metabolic pathways, followed by lipid metabolism and folate metabolism. This indicated that mainly small molecule metabolites were affected during pasteurization and freeze-drying, followed by lipids, but proteins and amino acids were not affected. High heat (>85 °C) altered physical and chemical properties of jenny milk proteins (Miao et al., 2020), thus freeze-drying could preserve the components of proteins and amino acids, which was reported in human, cow and camel milk (Blackshaw et al., 2022; Harizi et al., 2023; Zhu et al., 2020).

### 4.3 Essential and toxic trace elements

It was reported that mineral elements in jenny milk were affected by lactation stage, location, and breed (Garhwal et al., 2023; Ljubojević Pelić et al., 2025; Malacarne et al., 2019; Nayak et al., 2020), but the impacts of storage and processing remain unclear. The Co concentration in liquid milk from cows, goats, buffaloes, yaks, and camels in China ranges from 0.28 to 4.3 μg/kg (Yan, Niu, et al., 2022), while in pasteurized and sterilized milk the concentration is less than 50 μg/L (Alinezhad et al., 2024). In the present study, the Co concentrations in raw and pasteurized jenny milk were within the above ranges, although the concentration in FM was higher. Cobalt is an essential trace element for humans, and plays a key role in the formation of vitamin B_12_, with a dietary requirement of 3–5 μg/d (Genchi et al., 2023). Based on this estimate, daily intake of 30 g of freeze-dried jenny milk meets the daily requirement for Co; thus freeze-dried jenny milk powder is a good source of Co.

The Mn concentration ranges between 1 and 30 μg/L in breast milk (Klein et al., 2017), and 14 and 54 μg/L in horse milk (Fantuz et al., 2013). In the present study, the Mn concentration in jenny milk ranged between 13 and 25 μg/L, which was similar to the concentrations in breast and horse milk, but higher than in jenny milk (2.8 to 4.5 μg/L) reported by Fantuz et al. (2013). The main sources of Mn in milk are from drinking water and feed (Schmidt, 2021), but supplementing Mn in the diet did not affect the Mn concentration in jenny milk (Fantuz et al., 2013). We reasoned that Mn concentration in drinking water differed among regions and that may be the reason for differences between studies.

Concentrations of Cu, Fe, Zn, Se and I in jenny milk ranged between 73 and 123 μg/L, 81 and 9590 μg/L, 1330 and 3490 μg/L, 3.3 and 5.9 μg/L, and 40 and 103 μg/L, respectively (Fantuz et al., 2013; Ljubojević Pelić et al., 2025; Malacarne et al., 2019). The concentrations of these elements in the present fell in the ranges of previous studies.

This is the first report of Mo concentration in jenny milk. The Mo concentration in breast milk ranged between 0.1 and 25.9 μg/L, with a median of 3.18 μg/L (Yoshida et al., 2008) and of cow milk ranged between 33 and 71 μg/L, with an average of 50 μg/L (Zwierzchowski & Ametaj, 2019). In comparison, the Mo concentration in jenny milk ranged between 6.8 and 17.1 μg/L, which was similar to breast milk but lower than cow milk.

The concentrations of Co, Mn, Zn, Ni and Cr were relatively high in FM, while I was relatively high in PM. Due to the strict control over the phthalic acid esters (PAEs) such as DEHP, DEP, DBP, and DOP in food (Sokołowski et al., 2024), food-grade stainless steel (SUS304) containers and not polyethylene containers are recommended for milk storage and processing in China. The SUS304 steel contains large amounts of Cr (18% to 20%), and Ni (8.0% to 10.5%), and a small amount of Mn (China Iron and Steel Association, 2023). Since FM requires a long processing time and many technical procedures, we reasoned that the increase in Mn, Ni and Cr in FM could be related to the stainless-steel processing equipment. Based on the latest risk assessments, the Ni content in jenny milk did not exceed the maximum limit set by the EU infant formula/special medical use formula food (European Commission, 2024), and the level of Cr was also within the limit (Ministry of Health of the People's Republic of China, 2025). The reason for the increase in Co and Zn in FM and I in PM warrants further research.

The Pb, Cd, Hg and As are potentially toxic elements. The concentration of As in FM was higher than in RM, DM and PM, but the Hg did not differ among the 4 milk types. In China, the safety limit for Hg in liquid milk is 10 μg/kg, while the limit for As is 500 μg/kg in milk powder and 100 μg/kg in liquid milk (Ministry of Health of the People's Republic of China, 2025). The Chinese standard (Ministry of Health of the People's Republic of China, 2025) and European Union standard (Directorate-General for Health and Food Safety of European Commission, 2023) set the safety limit of Pb at 200 μg/kg in milk powder and 20 μg/kg in liquid milk, but no limit was set for Cd and Sn in dairy products. In the present study, the concentrations of Sn were detected in jenny milk firstly, and the concentrations of Pb, Hg, As and Cr of all jenny milk types were lower than the safety limits, with the concentration of Pb the in PM and DM reaching 70 to 82% of the safety limits. In milk and dairy products across the globe, the concentrations Cu, Cr, Zn, and Pb exceeded the allowable maximum concentrations in 94%, 67%, 62% and 46%, respectively, of the tested samples (Alinezhad et al., 2024). The main sources of toxic elements in milk are from feed, water, and milk containers (Ljubojević Pelić et al., 2024). Safety control measures should be strengthened during the production and processing of jenny milk to ensure food safety.

### 4.4 Fatty acid composition

Milk FA occur in both FFA and esterified forms, and their distributions are closely related to lipid stability, flavor development, and shelf-life of milk products (Wei et al., 2025). In the present study, the milk fat content did not differ among milk types, but the levels of free propionic, isobutyric, and isovaleric acids were higher in PM than in RM, and the levels of propionic, butyric, isovaleric, hexanoic, and caprylic acids, and total FSMCFA were higher in FM than in RM. These results suggest that storage and processing altered the free fatty acid fraction even when total fat content remained relatively stable.

The increase in FSMCFA in PM and FM may be associated with several processing-related factors, including enzymatic lipolysis, thermal effects, and mechanical disturbance during milk handling and processing. Heat treatment and storage can affect milk FFA, lipid oxidation, and lipid-derived volatile compounds (Zhang et al., 2022), while mechanical actions such as pumping, stirring, and freeze-drying can disturb the milk fat globule membrane and promote lipolysis (Woodhouse et al., 2025). In addition, lipases in milk can hydrolyze TG in milk to produce FFA, and these FFA can produce aldehydes, ketones, alcohols, esters, and lactones through metabolism, oxidation, and heat treatment and, thus, produce a lipolytic rancid odor and impair milk flavor (Tetra Pak, 2025). Similar to the findings in the present study, the FFA content in human milk increased slightly due to freeze-drying, and small amounts of terpenes, alcohols, and ketones were detected, but the total FA level was not affected (Blackshaw et al., 2022). Therefore, the increase in FSMCFA in PM and FM may have important implications for flavor stability of processed jenny milk.

The main MLCFA in jenny milk was palmitic, oleic, and linoleic acids, which was also reported in previous studies on jenny milk (Liang et al., 2025; Martemucci & D'Alessandro, 2012). The current study demonstrated that, compared with RM, storage and processing reduced the percentage of MCFA and increased the percentage of LCFA, while freeze-drying increased the concentrations and percentages of MUFAs, n-6 PUFA, and n-9 UFA. However, the increase in LCFA may reflect the relative reduction or loss of MCFA rather than an increase in LCFA. Possible mechanisms include the partial lipolysis of triacylglycerols to release the MCFA into the FFA fraction, volatility-related loss or transformation of SCFA during storage and heating, and greater retention of less volatile LCFA during processing. This interpretation is consistent with previous findings that heating and storage can modify milk FA profiles and lipid-derived volatile compounds (Ao et al., 2014).

The FA profile can affect the flavor (Kilic-Akyilmaz et al., 2022), antioxidant properties and shelf life of milk (Deeth, 2020). During the process from low-temperature freezing storage of raw jenny milk to the production of freeze-dried milk powder, the levels of FSMCFA and LCFA increased, but MCFA decreased. In particular, the increase in n-6 and n-9 FA can accelerate the oxidation of FA, shorten shelf life, and spoil the flavor in milk powder. Therefore, changes in milk fat should be of concern with thermal processing of jenny milk.

Consistent with these changes in FA composition, the correlation analysis further showed that the processing- and storage-related changes in milk FA profiles were related to changes in small-molecule composition, which possibly involved lipid oxidation, flavor precursor formation, and changes in the milk matrix. Of particular interest, 1-methyladenosine and oxoadipic acid were correlated positively with MCFA and LCFA, whereas differential amino acid-, aromatic compound-, carbohydrate-, and phospholipid-related metabolites were correlated negatively. The latter were correlated positively with SCFA and branched-chain FA. Some studies reported that milk lipid profiles, FA composition, and volatile compounds can be influenced by heat treatment, refrigeration, and storage conditions, thereby affecting lipid oxidation and flavor-related compound formation (Justyna et al., 2024; Yan et al., 2024; Zhang et al., 2022). Overall, our findings indicated that alterations in FA profile, including FSMCFA, MCFA, LCFA, and UFA, were associated closely with the metabolomic differences among raw, stored, pasteurized, and freeze-dried jenny milk.

From an industrial processing perspective, these results indicate that the quality of raw lipid should be considered a key in jenny milk processing. Rapid cooling after milking, reduction of storage time before processing, optimization of pasteurization conditions, and control of oxygen exposure and packaging during freeze-drying could reduce lipolysis and lipid oxidation. For freeze-dried jenny milk powder, oxygen-barrier or nitrogen-flushed packaging may be useful to protect UFA and maintain flavor stability.

## 5 Conclusions

The present study examined the longitudinal differences in proximate components, pH, essential and toxic trace elements, untargeted metabolomes, and FA of fresh raw, low-temperature stored, pasteurized, and freeze-dried jenny milk. Measurements were made in a commercial dairy and, consequently, the results are relevant to the jenny milk industry. We are aware that the number of samples were uneven and small for several measured variables in the present study, but the results revealed that: (1) neither cryopreservation nor heat treatment altered the proximate components of jenny milk; (2) contents of all detected elements were within safe health limits; (3) mainly non-lipid components in jenny milk were affected between milking and low-temperature storage. The pasteurized and lyophilized milk altered the FA profile, which reduced the percentage of MCFA and increased the percentages of LCFA, MUFA, n-6 PUFA, and n-9 UFA in lyophilized jenny milk, although most of the FA concentrations were not altered. Freeze-drying had little effect on protein and amino acid components. Therefore, after milking, attempts should be made to shorten the cooling and storage time, while the heat treatment should focus on the oxidation loss of unsaturated fatty acids. Further studies on more commercial dairies and with larger sample sizes are warranted to strengthen the results of this study.

## CRediT authorship contribution statement

**Le-tian Zhang:** Writing – original draft, Visualization, Data curation. **Ke Zhang:** Investigation, Data curation. **Allan Degen:** Writing – review & editing. **Qian Xu:** Software, Formal analysis. **Jiang-lin Xiong:** Writing – review & editing, Methodology. **Xiao-ling Zhou:** Writing – review & editing, Validation, Project administration, Funding acquisition, Conceptualization.

## Funding sources

This work was supported financially by the Bingtuan Science and Technology Program (2026YD015), Tianshan Talent Project (2024SNGGNT-BT04), and National Natural Science Foundation of China (32260844).

## Declaration of competing interest

The authors declare the following financial interests/personal relationships which may be considered as potential competing interests: Zhou Xiaoling reports financial support was provided by Xinjiang Production and Construction Corps. If there are other authors, they declare that they have no known competing financial interests or personal relationships that could have appeared to influence the work reported in this paper.

## Data Availability

The data that has been used is confidential.
