# Supplementary material for: Changes in milk composition of raw, frozen, pasteurized, and freeze-dried jenny milk from a farm-scale dairy
Source: Food Chem X. 2026 Jul 22;38:104240. doi: 10.1016/j.fochx.2026.104240 (PMC13444257; doi:10.1016/j.fochx.2026.104240)
Supplement: Supplementary file 1 [file mmc2.docx]

**Change in milk composition of raw, pasteurized, and freeze-dried jenny milk under farm scale dairy**

Le-tian Zhang^1^, Ke Zhang^1^, Allan Degen^2^, Qian Xu^3^, Jiang-lin Xiong^4^, Xiao-ling Zhou^1*^

^1^College of Animal Science and Technology, Tarim University, Key Laboratory of Livestock and Grass Resources Utilization around Tarim, Ministry of Agriculture and Rural Areas, Key Laboratory of Tarim Animal Husbandry Science and Technology, Xinjiang Production & Construction Corps, Alar, Xinjiang 843300, China

^2^Desert Animal Adaptations and Husbandry, Wyler Department of Dryland Agriculture, Blaustein ^3^Institutes for Desert Research, Ben-Gurion University of the Negev, Beer Sheva 8410500, Israel

College of Food Science and Technology, Tarim University, Alar, Xinjiang 843300, China

^4^Hubei Key Laboratory of Animal Nutrition and Feed Science, School of Animal Science and Nutrition Engineering, Wuhan Polytechnic University, Wuhan 430023, China

***Corresponding Author:** Xiao-ling Zhou. Address: College of Animal Science and Technology, Tarim University, Alar, Xinjiang 843300, China. Tel: 13899289376, E-mail: zxldky@126.com

## 1. Untargeted metabolomics analysis

### 1.1 HPLC conditions

All samples were analyzed in duplicate using liquid chromatography-mass spectrometry (LC/MS). One aliquot was analyzed using positive ion conditions and was eluted from a T3 column (Waters ACQUITY Premier HSS T3 Column 1.8 µm, 2.1 mm * 100 mm) using 0.1 % formic acid in water as solvent A and 0.1 % formic acid in acetonitrile as solvent B in the following gradient: 5 to 20 % for 2 min, increased to 60 % in the following 3 min, increased to 99 % for 1 min and held for 1.5 min, then returned to 5 % mobile phase B within 0.1 min, and held for 2.4 min. The analytical conditions were as follows: column temperature: 40°C; flow rate: 0.4 mL/min; and injection volume: 4 μL. Another aliquot used negative ion conditions with the same gradient as the positive mode.

### 1.2 MS conditions

Data acquisition used the information-dependent acquisition (IDA) mode (Analyst TF 1.7.1 Software, Sciex, Concord, ON, Canada). The source parameters were set as follows: ion source gas 1 (GAS1), 50 psi; ion source gas 2 (GAS2), 50 psi; curtain gas (CUR), 25 psi; temperature (TEM), 550°C; declustering potential (DP), 60 V, or -60 V in positive or negative modes, respectively; and ion spray voltage floating (ISVF), 5000 V or -4000 V in positive or negative modes, respectively. The TOF MS scan parameters were set as follows: mass range, 50-1000 Da; accumulation time, 200 ms; and dynamic background subtract, on. The product ion scan parameters were set as follows: mass range, 25-1000 Da; accumulation time, 40 ms; collision energy, 30 or -30 V in positive or negative modes, respectively; collision energy spread, 15; resolution, UNIT; charge state, 1 to 1; intensity, 100 cps; exclude isotopes within 4 Da; mass tolerance, 50 ppm; and maximum number of candidate ions to monitor per cycle, 18.

### 1.3 Data analysis

### 1.3.1 PCA analysis

Unsupervised principal component analysis (PCA) used statistics function prcomp in R software (www.r-project.org). The data were unit variance scaled before unsupervised PCA.

### 1.3.2 Hierarchical Cluster Analysis and Pearson Correlation Coefficients

The hierarchical cluster analysis (HCA) results of samples and metabolites are presented as heatmaps with dendrograms, while Pearson correlation coefficients (PCC) between samples were calculated by the cor function in R software and presented as only heatmaps. Both HCA and PCC used the R package ComplexHeatmap. For HCA, normalized signal intensities of metabolites (unit variance scaling) are visualized as a color spectrum.

### 1.3.3 Differential metabolites selected

For two-group analysis, differential metabolites were determined by variable importance in projection (VIP > 1) and *P*-value (*P*-value < 0.05, Student‘s t-test). For multi-group analysis, differential metabolites were determined by VIP (VIP > 1) and *P*-value (*P*-value < 0.05, ANOVA). VIP values were extracted from orthogonal partial least squares discriminant analysis (OPLS-DA), which also contained score plots and permutation plots, and were generated using R package MetaboAnalystR. The data were log transformed (log 2) and mean centered before OPLS-DA. A permutation test (200 permutations) was used to avoid overfitting.

### 1.3.4 KEGG annotation and enrichment analysis

Identified metabolites were annotated using KEGG Compound database (<http://www.kegg.jp/kegg/compound/>), and annotated metabolites were then mapped to the KEGG pathway database (<http://www.kegg.jp/kegg/pathway.html>).

**1.4. Data quality control**

The superimposed results of the total ion chromatogram (TIC) (Figure S1) from the mass spectrometry (MS) detection of quality control (QC) samples showed that the total ion current curves for metabolite detection had a high degree of overlap, indicating that both the retention time and peak intensity were consistent. This indicated that the MS had good signal stability when detecting the same sample at different times.

Figure S1 Total ion chromatogram of quality control **(**QC) samples

Quality control (QC) samples were subjected to Pearson correlation analysis (Figure S2). The higher the correlation of QC samples (the |r| is closer to 1), the better the stability of the entire detection process and the higher the data quality.

Figure S2 Pearson correlation analysis of quality control **(**QC) samples

(Note: The diagonal squares represent the names of QC samples; and the squares in the upper right corner of the diagonal are the Pearson correlation coefficients corresponding to the QC samples.)

Internal standards with known concentrations were added to the QC samples. A small difference in the response of the internal standards (Table S1) indicated that the detection process was more stable and the data quality was high.

Table S1 Stability of internal standards in QC samples

| Index | Q1 (Da) | Retention time (min) | CV |
| --- | --- | --- | --- |
| MWS04187 | 208.1146 | 2.36 | 0.0491 |
| MWS4243 | 198.0333 | 2.45 | 0.0594 |
| MWS1055 | 126.0616 | 4.40 | 0.0677 |
| MWS3085 | 278.9898 | 4.25 | 0.0716 |

The empirical cumulative distribution function (ECDF) was used to analyze the frequency of occurrence of the coefficient of variation (CV) of substances that were less than a reference value. The percentage of substances with a CV value less than 0.5 in QC samples was higher than 85% （Figure S3), which indicated that the experimental data were relatively stable. The percentage of substances with a CV value less than 0.3 in QC samples was higher than 75%, which indicated that the experimental data were very stable.

Figure S3 Distribution of coefficient of variation (CV) for each group of samples

For the validation of the OPLS-DA model, the closer the three indicators R2X, R2Y, and Q2 are to 1, the more stable and reliable the model (Figure S4). When Q^2^ > 0.5, the model can be considered valid, and when Q^2^ > 0.9, the model is excellent.

Figure S4 Validation of the OPLS-DA model

**2. Trace element testing method**

2.1 Sample pre-treatment

Five mL of sample were added to 10 mL of aqua regia in a jar with a tightened lid and placed into the digestion instrument. After predigesting at 120°C for 30 min, the sample was digested in a microwave according to the following procedures: heated to 130°C within 5 minutes, held for 3 min; heated to 150°C within 3 min, held for 10 min; heated to 180°C within 3 min, and held for 30 min. After cooling to 60°C, the sample was diluted to 25 mL with deionized water, and shaken for testing.

2.2 Inductively coupled plasma mass spectrometry (ICP-MS) conditions

The ICP-MS detection used the standard mode for element determination. The detection conditions were as follows: generator power: 1300 w; detector analog stage voltage: -2350; ion lens voltage: 6.00 V; nebulizer flow rate: 0.98 L/min; plasma torch cooling gas flow rate: 17.0 L/min; and auxiliary flow rate: 1.20 L/min.

The mixed element standard was from the National Nonferrous Metals Corporation (product number 23DA0210 and 23D71176, National Nonferrous Metals Standardization and Testing Center, Beijing, China). The mercury standard substance [product number GBW(E) 083186, product batch number B23090303] and the iodine standard substance (product number BW20017-100-W-20, product batch number B22110252) were from Tanmo Quality Inspection Technology Co., Ltd. (Changzhou, China).

## 3. Free fatty acids analysis

3.1 Chemicals and reagents

Methyl tert-butyl ether (MTBE) was purchased (CNW Technologies, Germany) and MilliQ water (Millipore, Bradford, USA) was used in all experiments. All standards were purchased (CNW, Beijing, China or Aladdin, Shanghai, China). The stock solutions of standards were prepared at a concentration of 1 mg/mL in MTBE and were stored at -20 °C. The stock solutions were diluted with MTBE to working solutions before analysis.

3.2 Sample preparation and extraction

Fifty μL of sample were placed in a 2 mL EP tube, vortexed and thawed. Then, 200 μL of phosphoric acid (0.5% v/v) solution were added to the EP tube and vortexed for 10 min. The suspension was ultrasonicated for 5 min at 4℃ and centrifuged at 15200 × *g* for 10 min at 4°C. Then, 100 μL of supernatant were added to 250 μL MTBE (containing internal standard) solution in a 1.5 mL centrifuge tube, vortexed for 3 min, ultrasonicated for 5 min and centrifuged at 15200 × *g* for 10 min at 4°C. The supernatant was used for GC-MS/MS analysis.

3.3 GC-MS/MS analysis

Agilent 7890B gas chromatograph coupled to a 7000D mass spectrometer with a DB-FFAP column (30 m length × 0.25 mm i.d. × 0.25 μm film thickness, J&W Scientific, USA) was employed for GC-MS/MS analysis of SCFAs. Helium was used as carrier gas, at a flow rate of 1.2 mL/min. Injection was made in the split mode with a split ratio of 5:1 and the injection volume was 1 μL. The oven temperature was held at 50°C for 1 min, raised to 220°C at a rate of 18°C/min and held for 5 min. All samples were analyzed in multiple reaction monitoring mode. The injector inlet and transfer line temperatures were 250 °C and 230 °C, respectively, ion source temperature was 230°C, and quad temperature was 150°C.

3.4 Qualitative and Quantitative Analysis

Qualitative analysis of mass spectrometry data used the database based on standard compounds. After obtaining the mass spectrometry data of different samples, the chromatographic peak areas of all target substances were quantified by a linear equation of standard compound (Table S2).

Table S2 Quantitative linear equations of standard compounds

| **Fatty acid** | **Retention time**  (min) | **Equation** | **R^2*^** | **LLOQ**(μg/mL) | **ULOQ**(μg/mL) |
| --- | --- | --- | --- | --- | --- |
| Acetic acid | 7.288 | y = 1.347 x + 0.412 | 0.9963 | 0.02 | 20 |
| Propionic acid | 7.917 | y = 0.996 x | 0.9951 | 0.02 | 20 |
| Isobutyric acid | 8.115 | y = 0.355 x | 0.9964 | 0.02 | 20 |
| Butyric acid | 8.544 | y = 1.597 x + 0.019 | 0.9958 | 0.02 | 20 |
| Isovaleric acid | 8.828 | y = 9.451x - 0.008 | 0.9924 | 0.02 | 20 |
| 2-Methylbutyric acid | 8.829 | y = 0.800 x + 0.103 | 0.9951 | 0.05 | 20 |
| Valeric acid | 9.279 | y = 10.508 x + 0.095 | 0.9934 | 0.05 | 20 |
| Isocaproic acid | 9.700 | y = 6.374 x - 0.162 | 0.9982 | 0.05 | 20 |
| Caproic acid | 9.962 | y = 9.799 x + 0.840 | 0.9910 | 0.02 | 20 |
| Isoheptanoic acid | 10.329 | y = 7.539 x - 0.104 | 0.9960 | 0.05 | 20 |
| 2-Ethylcaproic acid | 10.581 | y = 0.934 x + 0.040 | 0.9922 | 0.02 | 20 |
| Heptanoic acid | 10.610 | y = 0.799 x + 0.014 | 0.9978 | 0.05 | 20 |
| 3,5,5-Trimethylhexanoic acid | 10.787 | y = 0.266 x - 0.002 | 0.9910 | 0.05 | 20 |
| Octanoic acid | 11.296 | y = 0.340 x + 0.024 | 0.9968 | 0.02 | 20 |
| Nonanoic acid | 12.083 | y = 0.055 x + 0.052 | 0.9966 | 0.10 | 20 |
| Isodecanoic acid | 12.566 | y = 0.127 x - 0.002 | 0.9984 | 0.10 | 20 |
| Decanoic acid | 13.033 | y = 0.285 x + 0.004 | 0.9971 | 0.05 | 20 |

^*^R^2^ = Coefficient of determination；LLOQ and ULOQ = lower and upper limits of quantification, respectively.

## 4 Entire middle- and long-chain fatty acid analysis

**4.1 Chemicals and reagents**

HPLC-grade methyl tert-butyl ether (MTBE), methanol (MeOH) and n-hexane (Merck, Darmstadt, Germany), MilliQ water (Millipore, Bradford, USA), NaCl and phosphate (Sigma-Aldrich, St. Louis, MO, USA, and methanol solution of 15% boron trifluoride (RHAWN, Shanghai, China) and standards (Sigma-Aldrich, St. Louis, MO, USA) were used. The stock solutions of standards were prepared at the concentration of 1 mg/mL in MTBE, stored at -20℃ and diluted with MTBE to working solutions before analysis.

**4.2 Sample preparation and extraction**

After the sample was thawed, 0.05 mL were added to 150 µL MeOH, 200 μL MTBE, 50 μL 36% phosphoric acid/water (v/v, precooled at -20℃) in a tube, vortexed for 3 min, ultrasonated for 10 min and centrifuged at 15200 × *g* for 5 min at 4℃. Then 200 μL of supernatant were concentrated by a nitrogen blower until dry and 300 μL of 15% boron trifluoride methanol solution were added to the dried sample, vortexed for 3 min and kept in an incubator for 30 minutes at 60℃ to harvest the derivatization. Then 500 μL of n-hexane solution and 200 μL of saturated sodium chloride solution were added to the derivative solution and cooled to room temperature. The mixture was vortexed for 3 min, centrifuged at 15200 × *g* for 5 min at 4℃, and the upper n-hexane layer solution was transferred into a GC injection vial for analysis.

**4.3 GC and MS Conditions**

The sample derivants were analyzed using gas chromatography (Agilent 7890B, Santa Clara, CA, USA) coupled to triple quadrupole mass spectrometry (Agilent 7000D). The GC analytical conditions were as follows: the separation was conducted on a DB-5MS capillary column (30 m×0.25 mm×0.25 μm, Agilent) with high purity helium (purity >99.999%) as a carrier gas. Heating started at 40℃ and held for 2 min, increased to 200℃ at a rate of 30℃/min and held for 1 min, increased to 240℃ at a rate of 10℃/min and held for 1 min, and increased to 285℃ at a rate of 5℃/min and held for 3 min. The flow rate was set at 1 mL/min, injection volume was set at 1 μL with splitless, and the inlet temperature was set at 250℃.

The mass spectrometry scanning mode used ion monitoring mode (SIM). The mass spectrometry parameters were set as: temperature and ionization voltage of electron ionization source (EI source) was 230℃ and 70eV, respectively; transmission line and quadrupole temperature was 280℃ and 150℃, respectively, and sample data were acquired in SIM mode after solvent delay for 4 min.

**4.4** Qualitative and Quantitative Analysis

Standard solutions prepared with the concentrations of 0.01, 0.02, 0.05, 0.1, 0.2, 0.5, 1, 2, 5, 10, 20, and 50 μg/mL, and the chromatographic peak area data corresponding to each concentration of the standard were obtained. The standard curve for different substances was constructed using the standard concentration (concentration ratio) as the x-value and the peak area (area ratio) as the y-value. The linear equations and correlation coefficients of the standard substances detected are presented in Table S3.

Table S3 The linear equations of entire middle- and long-chain fatty acids

| **Fatty acid** | **Retention time (min)** | **Equation** | **R^2*^** | **LLOQ**  (μg/mL) | **ULOQ**  (μg/mL) |
| --- | --- | --- | --- | --- | --- |
| hexanoic acid | 4.163 | y = 5159747.370 x + 3869.367 | 0.9995 | 0.01 | 10 |
| octanoic acid | 5.384 | y = 51315.099 x - 20.559 | 0.9997 | 0.01 | 10 |
| nonanoic acid | 5.928 | y = 95011.200 x - 190.809 | 0.9996 | 0.01 | 10 |
| decanoic acid | 6.468 | y = 148286.145 x - 291.635 | 0.9992 | 0.01 | 10 |
| cis-10-undecanoic acid | 6.978 | y = 1651194.338 x - 33397.660 | 0.9980 | 0.05 | 10 |
| Undecanoic acid | 7.020 | y = 4908363.020 x - 6405.735 | 0.9990 | 0.01 | 5 |
| lauric acid | 7.601 | y = 297091.328 x - 473.229 | 0.9992 | 0.01 | 10 |
| tridecanoic acid | 8.246 | y = 376208.053 x - 854.0380 | 0.9994 | 0.01 | 10 |
| myristoleic acid | 8.905 | y = 98731.898 x - 138.675 | 0.9994 | 0.01 | 10 |
| myristic acid | 8.990 | y = 464530.640 x - 1459.876 | 0.9995 | 0.01 | 10 |
| cis-10-pentadecenoic acid | 9.755 | y = 95370.796 x + 77.029 | 0.9993 | 0.01 | 10 |
| pentadecanoic acid | 9.847 | y = 555573.868 x - 2642.769 | 0.9997 | 0.01 | 10 |
| cis-9-palmitoleic acid | 10.640 | y = 94445.303x + 73.405 | 0.9973 | 0.01 | 10 |
| trans-9-palmitelaidic acid | 10.683 | y = 114796.380x + 815.669 | 0.9993 | 0.01 | 10 |
| palmitic acid | 10.846 | y = 593543.537x - 3409.876 | 0.9992 | 0.02 | 10 |
| cis-10-heptadecanoic acid | 11.745 | y = 92051.321x - 308.835 | 0.9984 | 0.01 | 30 |
| trans-10-heptadecenoic acid | 11.802 | y = 93262.918 x - 150.744 | 0.9977 | 0.01 | 30 |
| heptadecanoic acid | 11.979 | y = 673709.585x - 4834.892 | 0.9990 | 0.01 | 10 |
| γ-linolenic acid | 12.694 | y = 94038.203x - 2936.250 | 0.9971 | 0.05 | 5 |
| linoleic acid | 12.928 | y = 223566.197x - 8581.506 | 0.9986 | 0.05 | 20 |
| α-linolenic acid | 13.027 | y = 84820.649x - 640.098 | 0.9986 | 0.01 | 30 |
| cis-9-octadecenoic acid | 13.027 | y = 87381.273x - 481.206 | 0.9972 | 0.01 | 30 |
| linolelaidic acid | 13.049 | y = 265645.774x - 2623.038 | 0.9984 | 0.01 | 10 |
| trans-9-octadecenoic acid | 13.119 | y = 100471.904x - 1132.749 | 0.9995 | 0.01 | 30 |
| trans-Vaccenic acid | 13.176 | y = 99874.734x - 719.139 | 0.9976 | 0.02 | 10 |
| stearic acid | 13.417 | y = 708657.682x - 5720.656 | 0.9991 | 0.01 | 30 |
| cis-10-carboenoic acid | 14.614 | y = 74058.909x - 780.943 | 0.9983 | 0.05 | 20 |
| nonadecylic acid | 14.975 | y = 690070.125x - 6502.664 | 0.9991 | 0.01 | 30 |
| hexadecanedioic acid | 15.308 | y = 493928.249x - 16692.600 | 0.9919 | 0.05 | 2 |
| arachidonic acid | 15.379 | y = 7540.757x - 67.817 | 0.9933 | 0.02 | 2 |
| cis-5,8,11,14,17-eicosapentaenoic acid | 15.464 | y = 25962.968x - 808.508 | 0.9916 | 0.05 | 2 |
| cis-8,11,14-eicosatrienoic acid | 15.627 | y = 81554.894x - 2472.398 | 0.9942 | 0.05 | 2 |
| cis-11,14-eicosadienoic acid | 15.896 | y = 163645.361x - 6059.720 | 0.9954 | 0.05 | 5 |
| cis-11-eicosenoic acid | 15.974 | y = 58593.016x - 1472.721 | 0.9986 | 0.05 | 20 |
| cis-11,14,17-eicosatrienoic acid | 15.995 | y = 82655.718x - 4035.330 | 0.9984 | 0.05 | 30 |
| trans-11-eicosenoic acid | 16.066 | y = 63740.486x - 345.053 | 0.9936 | 0.01 | 2 |
| arachidic acid | 16.349 | y = 602772.209x - 15404.525 | 0.9963 | 0.05 | 2 |
| heneicosanoic acid | 17.830 | y = 551660.456x - 20579.045 | 0.9984 | 0.05 | 20 |
| cis-4,7,10,13,16,19-docosahexaenoic acid | 18.064 | y = 1513807.678x - 447515.700 | 0.9982 | 0.5 | 20 |
| cis-7,10,13,16-docosatetraenoic acid | 18.149 | y = 9737.700x - 625.071 | 0.9938 | 0.1 | 5 |
| cis-7,10,13,16,19-docosapentaenoic acid | 18.220 | y = 2128.395x - 394.787 | 0.9987 | 0.2 | 20 |
| cis-13,16-docosadienoic acid | 18.588 | y = 99190.165x - 1073.772 | 0.9946 | 0.01 | 5 |
| erucic acid | 18.637 | y = 31604.137x - 985.741 | 0.9972 | 0.05 | 5 |
| trans-13-docosenoic acid | 18.708 | y = 27735.618x - 169.656 | 0.9932 | 0.01 | 2 |
| behenic acid | 18.914 | y = 401776.171x - 2907.334 | 0.9929 | 0.01 | 2 |
| tricosanoic acid | 20.132 | y = 318985.218x - 2856.752 | 0.9953 | 0.01 | 5 |
| nervonic acid | 20.940 | y = 13061.168x - 133.031 | 0.9981 | 0.01 | 5 |
| lignoceric acid | 21.180 | y = 207616.675x - 785.444 | 0.9940 | 0.01 | 2 |

^*^R^2^ = Coefficient of determination; LLOQ and ULOQ = lower and upper limits of quantification, respectively.
